# Studies on the prevalence of Hepatitis C virus infection in diabetic patients attending a tertiary health-care facility South-west Nigeria

**DOI:** 10.1186/s12879-020-05388-7

**Published:** 2020-09-09

**Authors:** James A. Ndako, Akinyomade O. Owolabi, Joseph A. Olisa, Jeremiah A. Akinwumi, Victor T. Dojumo, Oludolapo Olatinsu, Blessing A. Adebayo

**Affiliations:** 1 Department of Microbiology, Landmark University, Omu-aran, Nigeria; 2 Department of Medical Services, Landmark University Medical Center, LMU, Omu-aran, Nigeria; 3 Department of Medical Laboratory Services, Landmark University Medical Center, LMU, Omu-aran, Nigeria

**Keywords:** HCV, Type 2 diabetes mellitus (T2DM), Patients

## Abstract

**Background:**

Hepatitis C virus (HCV) infection and type 2 diabetes mellitus (T2DM) are two major public health problems associated with increasing complications and mortality rates worldwide. The objective of this study is to evaluate the prevalence of hepatitis C virus (HCV) infection in diabetic patients and to investigate the influence of several epidemiological and clinical factors on HCV infection.

**Method:**

A total number of one hundred and eighty diabetic patients were recruited for this study. Consented subjects made up of 71(39.4%) males and 109(60.56%) females were recruited for the study. While one-Hundred (100) Non-Diabetics (Controls) were also recruited for the study. Structured questionnaires were administered to the consented participants to obtain relevant data. Sera samples were assayed for antibodies to HCV using an enzyme linked immunosorbent assay [Inteco Diagnostic Limited]. ELISA technique.

**Result:**

Overall prevalence of HCV infection among diabetes patients assayed was 13.3% out of which 8(11.3%) was obtained from the male subjects compared to 16 (14.7%) seropositivity recorded among the females (*P* = 0.511; *P* > 0.05). Considering age distribution, Subjects aged 41–50 years recorded, 9 (22.5%) positivity (*P* = 0.238; *P* > 0.05).Considering educational status of subjects screened, 22 (14.9%) positivity was rescored among subjects who have attained tertiary status of education.(*P* = 0.574;*P* > 0.05).Risk factors considered showed that, 7 (18.9%) seropositive subject were alcoholic consumers(*P* value = 0.2621;*P* > 0.05) while 5 (8.9%) recorded history of sharing sharp objects *P* = 0.2427;P > 0.05).

**Conclusion:**

Our study shows a slightly higher prevalence of hepatitis C infection in type 2 diabetics. This call for urgent routine screening exercise among diabetic patients for HCV infection. This study also emphasizes the need for public enlightenment on the association between HCV infection and T2DM, to avert possible complications among diabetic patients.

## Background

Hepatitis is the inflammation of the liver commonly caused by a viral infection which can either be acute or chronic, symptomatic or asymptomatic [1]. This viral infection can lead to liver damage and further complications due to various causes such as heavy intake of alcohol, toxin ingestion and certain suppressive medical conditions [2].Hepatitis C virus (HCV) infection is a public health concern affecting more than 170 million people worldwide [3] [4]. HCV is a positive, single-stranded RNA virus in the Flaviviridae family. HCV is most efficiently transmitted through transfusion of infected blood, transplantation of infected organs, mother-to-child transmission during childbirth and interaction with infected blood or body fluids of an infected person [5].Most cases of Liver transplant mainly occurs as a result of HCV infection progressing to chronic infections [6].

Hepatitis C virus (HCV) has been identified as one of the leading causes of chronic liver disease with serious sequel as the end stage of cirrhosis and liver cancer [7].Hepatitis C has clearly been demonstrated to be a precipitating factor for diabetes but only in patients with risk factors to develop such. People with hepatitis C virus (HCV) infection appear to be at increased risk of developing type 2 diabetes [8].Diabetes mellitus is a chronic disease of metabolism causing abnormal glucose homeostasis [9].

Diabetes Mellitus, commonly referred to as diabetes is an impairment in which the body is unable to process glucose leading to reduction in the blood sugar level. Diabetes is characterized by high blood glucose level(hyperglycemia) with problems of fat, protein, and carbohydrate metabolism due to the body’s inability to secrete/act on insulin over a long period [10]. Diabetic status of individuals are divided into two; namely Type 1 diabetes which occurs mostly in children and adolescents and Type 2 diabetes which occurs mostly in adults, [11].

Knobler, et al. (2000) reports an increase in DM type-2 before the development of advanced liver cirrhosis [12]. Previous Research found that, after excluding chronic hepatitis C patients who received previous interferon treatment, higher fibrotic stages in liver histology and family history of DM were closely associated with higher prevalence of DM and impaired fasting glucose in patients with chronic hepatitis C [13].HCV infection and type 2 diabetes mellitus are two chronic conditions which contribute to a significant morbidity and mortality. A higher prevalence of type 2 diabetes mellitus has been observed in the developed world (2 to 9.4%) among patients with HCV infection than in those with other forms of chronic hepatitis [14, 15].

Type 2 diabetes is a debilitating disease condition, while the co-infection of type 2 diabetes and HCV has been established to worsen this condition, hence it has become very necessary for a screening exercise to determine the prevalence rate of HCV among diabetic patients at our location of study, so as to increase awareness of the populace and health practitioners on the dangers of the co-infectious status of this virus among diabetics.

## Method

### Study area and population

The study was conducted at the Federal Teaching Hospital Ido-Ekiti which is a tertiary health institution. The study population comprised of randomly selected confirmed Diabetic patients attending the outpatient department of the Teaching Hospital.

### Ethical permit and consent

A proposal of the project was submitted to the Ethical Review Committee of the Federal Teaching Hospital Ido-Ekiti. Where ethical permit was sought for and obtained with protocol number: ERC/2018/02/27/103B.

#### Inclusion and Exclusion criteria

Individuals confirmed for type 2 diabetes mellitus diabetes were recruited for the study. Persons who showed no interest in the study and are not diabetic were excluded from the study.

### Questionnaire and sample size

The sample size used in this research work was obtained from One–Hundred and eighty (180) volunteering confirmed diabetics, while One hundred (100) samples were also obtained from non-diabetic subjects to serve as control. All subjects recruited were informed about the study and their consents obtained. Well-structured questionnaires developed for this study were used to collect demographic data and other pertinent information (Additional file).

### Sample collection and processing

Three-Five [3–5] ml of blood were collected from both diabetic (Cases) and control subjects aseptically and according to standard procedure. Blood samples were allowed to clot at room temperature undisturbed, thereafter Sera obtained were dispensed into a clean, dry cryovial and stored at − 20 °C prior use. The sera were screened for antibodies to HCV using ELISA kits (Fortress Diagnostic Limited). Standard procedures were strictly adhered during the assay process.

### Data analysis

Filled questionnaires were crosschecked manually for correct data entry. The data were analyzed using the SPSS software package, Chi-square test was used to compare several Variables while the critical level for statistical significance was set at *P* = 5% (0.05).

#### Sample processing

Sera samples obtained were screened for HCV antibodies using ELISA technique according to the manufactures manual. Serum ALT was also assayed for, following the procedure detailed out in the kit used. Hepatitis C ELISA and ALT kits were stored in the refrigerator at 4 °C prior to use. Serum samples were analyzed at Landmark University Medical Laboratory.

## Results

The total sera samples screened comprises of 71 (39.4%) Males and 109 (60.56%) Females (Table 1).Positive samples obtained showed that, 8(4.4%) were obtained from the Male diabetic patients while the female subjects recorded 16(8.9%) positivity for HCV, (Tables 2 and 3).The age distribution of the subjects analyzed for the test ranged from 0 to 20, 21–30, 31–40, 41–50, 51–60 and 60–100 years. Of the 180 serum samples analyzed for HCV, 24 (13.3%) samples tested positive while 156 (86.7%) samples tested negative.

**Table 1:** Distribution of Hepatitis C Virus among diabetic subjects based on Gender

Gender	Total number of samples examined (%)	Number of Positive samples (%)	Number of Negative samples (%)
Male	71(39.4)	8(4.4)	63(35.0)
Female	109(60.6)	16(8.9)	93(51.7)
**Total**	**180(100.0)**	**24(13.3)**	**156(86.7)**

Chi square (x^2^) = 0.433; df = 1; *P* value = 0.511

**Table 2:** Distribution of sera samples assayed among Diabetic subjects screened

Total number of samples	Number of positive samples	Number of negative samples
180	24 (13.3%)	156 (86.7%)

**Table 3:** Distribution of HCV among Non-Diabetics (Control Subjects) based on gender

SEX	Number tested	HCV	
		Positive %	Negative%
Male	48	4(4%)	44 (44%)
Female	52	5 (5%)	47 (47%)
Total	100	9 (9%)	91 (91%)

*P* = 0.739; *P* > 0.05

Table 4 showed the age group of individuals tested, between 0 and 20 years, 20 (11.1%) individuals were tested yielding 0 (0.0%) which indicates 20 (100.0%) negative to HCV. For Subjects aged 21–30 years, 20 (11.1%) were screened out of which 2 (10.0%) showed positivity for HCV infection with 18 (90.0%) negative to HCV. Subjects aged 31–40 years, recorded 6(16.2%) positivity. Subjects aged 41–50 recorded 9 (22.5%) positivity correspondingly, subjects aged 51–60 years, recorded 3 (12.5%) positivity to HCV infection. Interestingly subjects aged 61–100 years, recorded 4 (10.3%) positivity.

**Table 4:** Distribution of Hepatitis C Virus based on Age of subjects screened

Age	Total number of samples examined (%)	Number of Positive samples (%)	Number of Negative samples (%)
0–20	20 (11.1)	0 (0.0)	20 (11.1)
21–30	20 (11.1)	2 (1.1)	18 (10.0)
31–40	37 (20.6)	6 (3.3)	31 (17.2)
41–50	40 (22.2)	9 (5.0)	31 (17.2)
51–60	24 (13.3)	3 (1.7)	21 (11.7)
60–100	39 (21.7)	4 (2.2)	35 (19.4)
**Total**	**180 (100.0)**	**24 (13.3)**	**156 (86.6)**

Chi square (x^2^) = 6.778; df = 5; *P* value = 0.238

Table 5 showed distribution of subjects based on marital status.130 (72.2%) married subjects were screened. 20 (15.4%) recorded positivity compared to 4 (9.8%) recorded among the single subjects.

**Table 5:** Distribution of Hepatitis C Virus According To Marital Status

Marital Status	Total number of samples examined (%)	Number of Positive samples (%)	Number of Negative samples (%)
Single	41 (22.8)	4 (2.2)	37 (20.6)
Married	130 (72.2)	20 (11.1)	110 (61.1)
Divorced	9 (5.0)	0 (0.0)	9 (5.0)
**Total**	**180 (100.0)**	**24 (13.3)**	**156 (86.7)**

Chi square (x2) = 2.312; df = 2; *P* value = 0.315

Table 6, showed the educational background of the subjects screened. Subjects with secondary education status recorded 2(8.0%) positivity compared to subjects with tertiary level of education recording 22 (14.9%) positivity.

**Table 6:** Distribution of Hepatitis C Virus According To Educational Background

Education	Total number of samples examined (%)	Number of Positive samples (%)	Number of Negative samples (%)
Primary	6 (3.3)	0 (0.0)	6 (3.3)
Secondary	25 (13.9)	2 (1.1)	23 (12.8)
Tertiary	148 (82.2)	22 (12.2)	126 (70.0)
No Education	1 (0.6)	0 (0.0)	1 (0.6)
**Total**	**180 (100.0)**	**24 (13.3)**	**156 (86.7)**

Chi square (x^2^) = 1.993; df = 3; *P* value = 0.574

Table 7, showed the demographic factor of the individuals was among which the traders recorded 7 (10.9%) positivity while Civil servants screened recorded 14 (21.9%) positivity.

**Table 7:** Distribution of Hepatitis C Virus based on Occupation of subjects screened

Occupation	Total number of samples examined (%)	Number Positive samples (%)	Number of Negative samples (%)
Trading	64 (35.6)	7 (3.9)	57 (31.7)
Civil Servant	64 (35.6)	14 (7.8)	50 (27.8)
Industry	18 (10.0)	2 (1.1)	16 (8.9)
Student	34 (18.9)	1 (0.6)	33 (18.3)
**Total**	**180 (100.0)**	**24 (13.3)**	**156 (86.7)**

Chi square (x^2^) = 7.613; df = 3; *P* value = 0.055

Table 8, Distribution of subjects screened based on clinical risk factors of subjects with history of blood transfusion recorded 1(6.7%) subjects with history of previously blood donation recorded 3 (23.1%) positivity for HCV infection. Subjects with history of care for a hepatitis patient resulted to 6 (15.8%) positivity.

**Table 8:** Distribution of Hepatitis C Virus based on Clinical Risk Factors

Risk Factor	Number of Positive Samples	Number of Negative Samples	Total Number of Samples Examined	Chi-Square
**Blood Transfusion**				
Positive	1 (0.6%)	14 (7.8%)	15 (8.3%)	**0.629**
Negative	23 (12.8%)	142 (78.9%)	165 (91.7%)	**df = 1**
**Total**	**24 (13.3%)**	**156 (86.7%)**	**180 (100.0%)**	**P value = 0.4276**
**Blood Donation**				
Positive	3 (1.6%)	10 (5.6%)	13 (7.2%)	**1.151**
Negative	21 (11.7%)	146 (81.1%)	167 (92.7%)	**df = 1**
**Total**	**24 (13.3%)**	**156 (86.7%)**	**180 (100.0%)**	**P value = 0.2833**
**History of Care for an Hepatitis C patient**				
Positive	6 (3.3%)	32 (17.8%)	38 (21.1%)	**0.251**
Negative	18 (10.0%)	124 (68.9%)	142 (78.9%)	**df = 1**
**Total**	**24 (13.3%)**	**156 (86.7%)**	**180 (100.0%)**	**P value = 0.6160**

Table 9 showed distribution of subjects based on social lifestyles, Subjects with history of alcohol consumption recorded 7 (18.9%) positivity while subjects with tribal marks or tattoos recorded 4 (21.1%) positivity subjects with history of sharing unsterilized equipment yielded 5 (8.9%) positivity to HCV infection. Interestingly, subjects with multiple sexual partners recorded 3 (42.9%) positivity to HCV infection.

**Table 9:** Distribution based on lifestyle-risk factors of Subjects Screened

Risk Factor	Response	Number of Positive Samples	Number of Negative Samples	Total Number of Samples Examined	Chi-Square
**Alcohol Consumption**	Positive	7 (3.9%)	30 (16.7%)	37 (20.6%)	**1.257**
	Negative	17 (9.4%)	126 (70.0%)	143 (79.4%)	**df = 1**
	**Total**	**24 (13.3%)**	**156 (86.7%)**	**180 (100.0%)**	**Pvalue = 0.2621**
**Tribal Marks and Tattoos**	Positive	4 (2.2%)	15 (8.3%)	19 (10.6%)	**1.095**
	Negative	20 (11.1%)	141 (78.4%)	161 (89.4%)	**df = 1**
	**Total**	**24 (13.3%)**	**156 (86.7%)**	**180 (100.0%)**	**Pvalue = 0.2953**
**Sharing of Unsterilized equipment**	Positive	5 (2.8%)	51 (28.3%)	56 (31.1%)	**1.365**
	Negative	19 (10.6%)	105 (58.3%)	124 (68.9%)	**df = 1**
	**Total**	**24 (13.4%)**	**156 (86.6%)**	**180 (100.0%)**	**Pvalue = 0.2427**
**Multiple sexual partners**	Positive	3 (1.7%)	4 (2.2%)	7 (3.9%)	**5.494**
	Negative	21 (11.7%)	152 (84.4%)	173 (96.1%)	**df = 1**
	**Total**	**24 (13.4%)**	**156 (86.6%)**	**180 (100.0%)**	**Pvalue = 0.0191**

Table 10, showed the distribution of subjects based on family history; subjects with previous history of hepatitis C virus yielded 1(0.6%) positivity; while those with family history of diabetes tested 12 (6.7.0%) positivity. Individuals with family members infected with hepatitis C virus yielded 3 (1.7%) positivity.

**Table 10:** Distribution of Hepatitis C Virus According To Family History

Risk Factor	Number of Positive Samples	Number of Negative Samples	Total Number of Samples Examined	Chi-Square
**Previous Record of HCV Infection**				
Positive	1 (0.6%)	4 (2.2%)	5 (2.8%)	**0.198**
Negative	23 (12.8%)	152 (84.4%)	175 (97.2%)	**df = 1**
**Total**	**24 (13.4%)**	**156 (86.6%)**	**180 (100.0%)**	**Pvalue = 0.6565**
**History of Diabetes by Family Members**				
Positive	12 (6.7%)	88 (48.9%)	100 (55.6%)	**0.346**
Negative	12 (6.7%)	68 (37.7%)	80 (44.4%)	**df = 1**
**Total**	**24 (13.4%)**	**156 (86.6%)**	**180 (100.0%)**	**Pvalue = 0.5563**
**History of infection with Hepatitis by Family Members**				
Positive	3 (1.7%)	29 (16.1%)	32 (17.8%)	**0.528**
Negative	21 (11.6%)	127 (70.6%)	148 (82.2%)	**df = 1**
**Total**	**24 (13.3%)**	**156 (86.7%)**	**180 (100.0%)**	**Pvalue = 0.4676**

## Discussion

The present study found a slightly higher prevalence rate of 13.3% among Type 2 Diabetes Mellitus (T2DM) subjects, compared to the global prevalence rate of around (3%) among the general population studied earlier. (Tables 2 and 3).The results obtained in this study compares with the work of Nwokediuko et al., [16] in Enugu where HCV occurrence rate among diabetic patients was found to be 14.0%. Although, Ejele et al.*,* [17] and Balogun et al., [18], obtained a prevalence of 3.0% in Niger Delta region and 0.0% in Ibadan which is lower than the result obtained in this study. Similar study conducted among diabetics by Ndako et al., [19] showed 11.0% prevalence in Jos which is also lower than the result obtained in this study, while a prevalence of 5.0% was recorded in a study carried out among diabetes patients at UITH, Ndako et al.*,* [20] while 5.7% prevalence was reported from India Demitrost et al.*,* [21] which is lower compared to the findings obtained in this study.However, Gray et al.*,* [22] was the first to show a higher prevalence of HCV infection in T2DM patients with a prevalence of 8% among Asian patients. Differences in the incidence rate of HCV results obtained from various regions globally depict geographical diversity. The variation in these occurrence rates can be ascribed to exposure to various risk factors which are capable of enhancing the spread and transmission of this virus among individuals [20, 21].

**Fig. 1:**
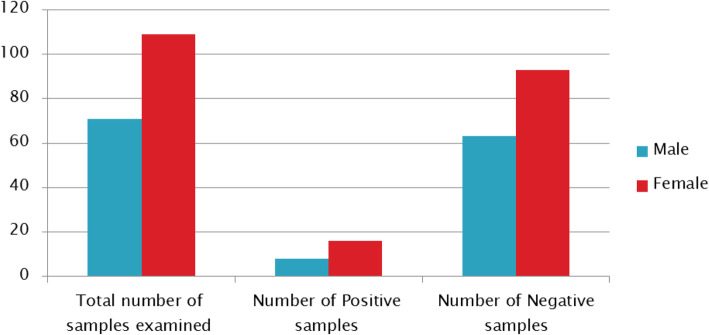
Distribution of Hepatitis C Virus according to Gender among diabetic subjects screened

The Prevalence rate of HCV infection among males, recorded 8(4.4%) while the female subjects had 14(8.9%) Seropositivity (Fig. 1). This finding agrees with the result obtained from a similar work by Ndako et al., [19] and Gacche et al., [23] where the incidence of anti-HCV among diabetic female were higher compared to the male subjects.Increased rate of occurrence in females could be attributed to various risk factors for HCV infection, which was quite evident from the life style and history of the individuals recruited for this study [19, 23].

**Fig. 2:**
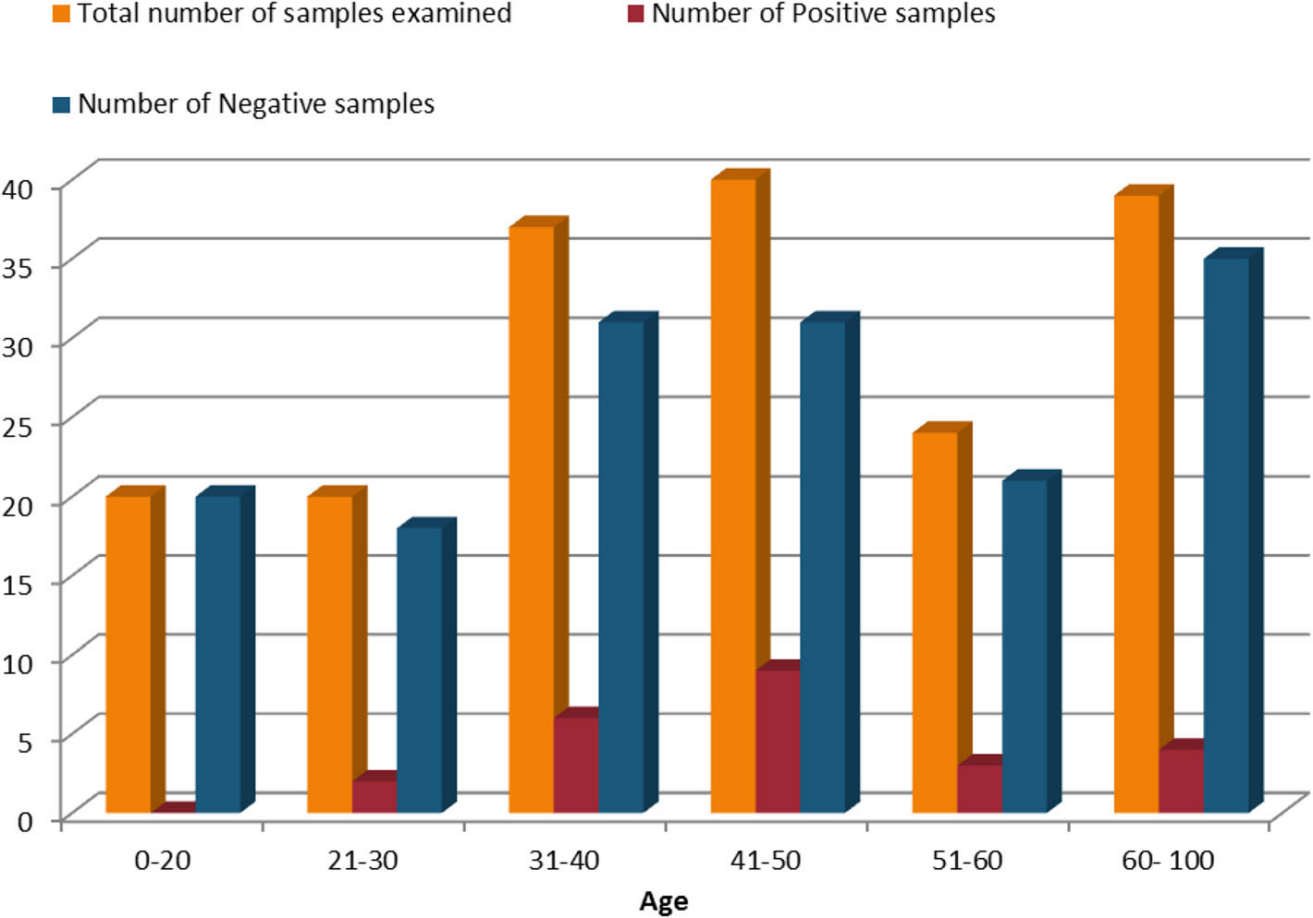
Distribution of Hepatitis C Virus according to Age

A higher prevalence rate of 9(5.0%) was observed amongst subjects aged 41–50; (Table 4; Fig. 2), this result is in accordance with the findings of Tessema et al., [24], where the sero-prevalence of HCV increases as age of participants increased and it was significantly higher in the age group of 41–50 years, which is almost similar to the results obtained by Klevens et al., [25] which showed a higher incidence rate among subjects aged 35–44,which also concurs with the result obtained in this study. The high seropositivity observed in older age group could be attributed to possible differences in social practices, parenteral exposures, decline in physical mobility and a reduced rate of medical examination compared to younger individuals thus increasing chances of transmission of infection, [26].

**Fig. 3:**
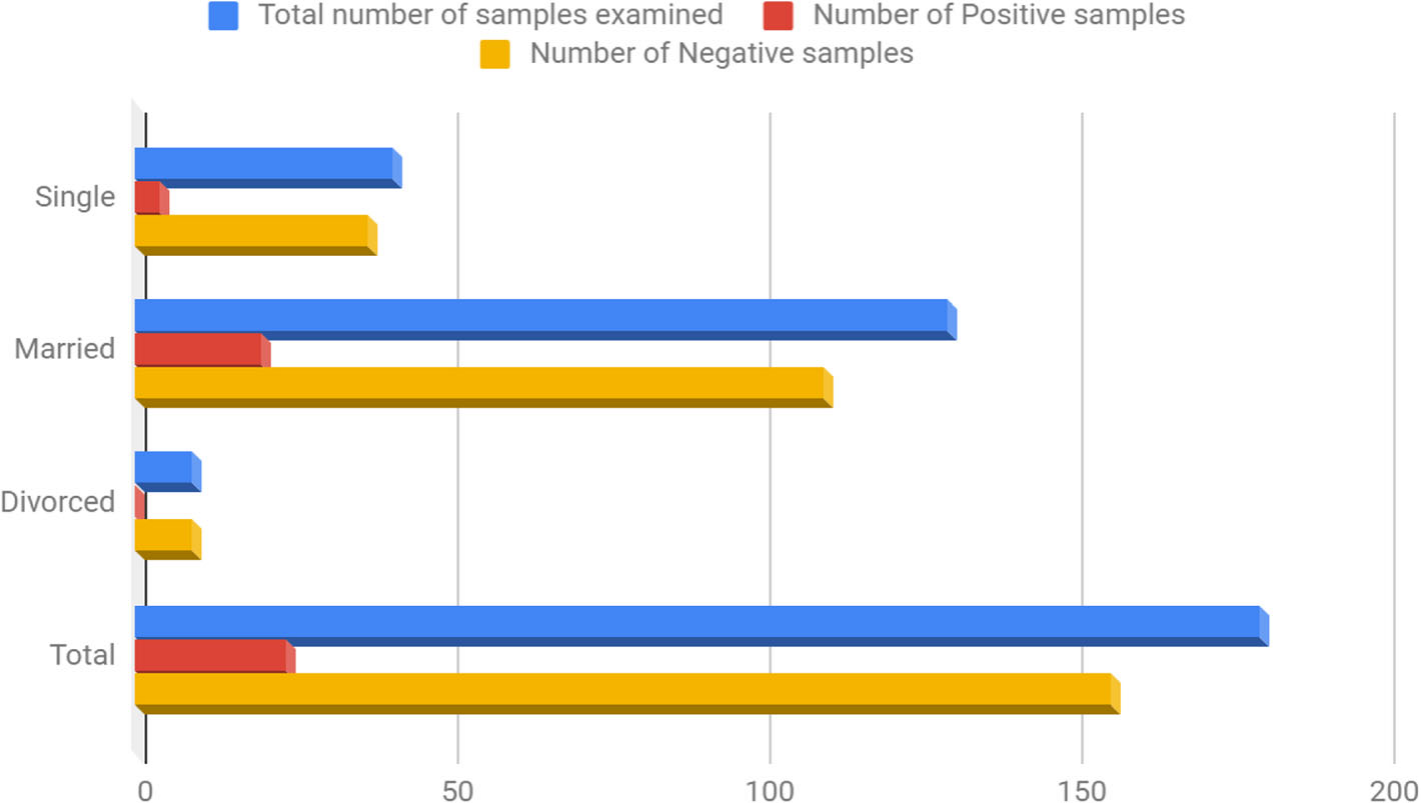
Distribution of Hepatitis C Virus according to marital status

The result obtained in this study showed that subjects with history of multiple sexual partners contributed immensely to the spread of HCV;(Table 5; Fig. 3) however, it was observed that the prevalence of HCV was higher among married subjects (15.4%), similar study carried out by Qureshi et al., [27] equally recorded a higher prevalence among married subjects. This is findings could be as a result of increased exposure to several risk factors to this infectious agent Simo et al., [28].

**Fig. 4:**
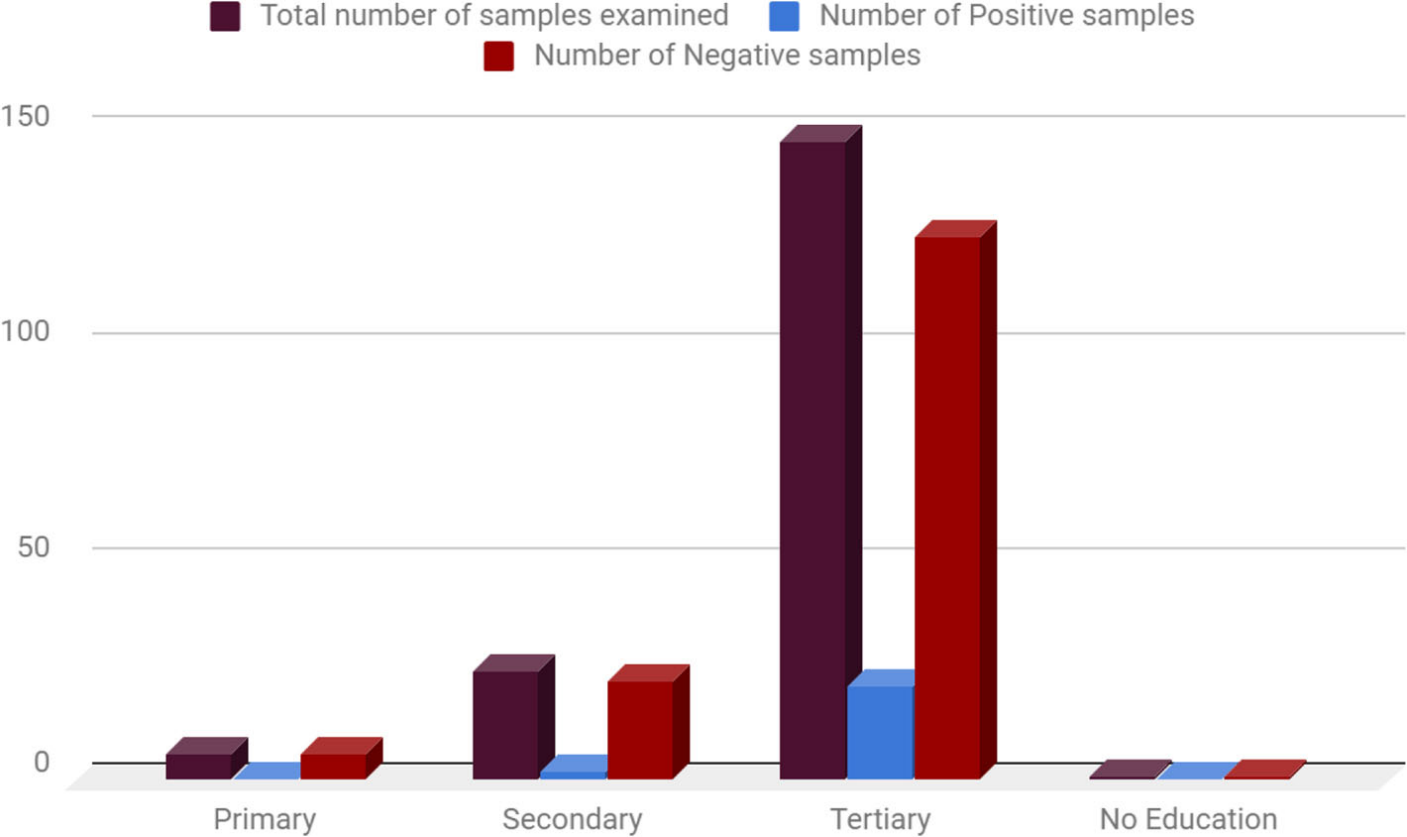
Distribution of Hepatitis C Virus according to Educational background

**Fig. 5:**
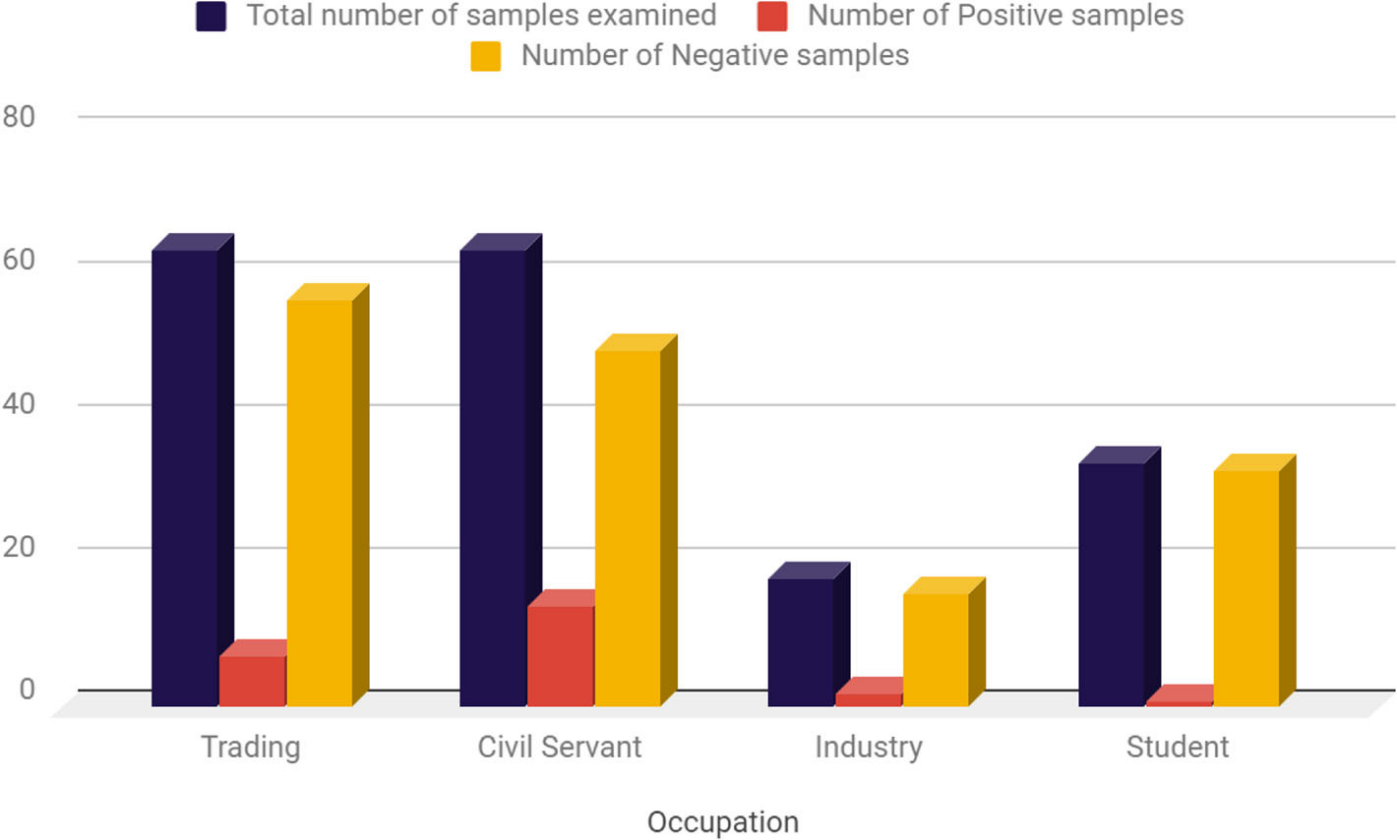
Distribution of Hepatitis C Virus according to occupation of Subjects screened

Educational background of the subjects screened showed that individuals with secondary education status recorded 2(8.0%) positivity compared to subjects with tertiary level of education recording 22 (14.9%) positivity, (Table 6; Fig. 4). Based on the various occupation of subjects screened in this study, it was found that individuals with trading as a profession recorded 7 (10.9%) positivity compared to Civil servants who recorded 14 (21.9%) positivity (Fig. 5). This study did not observed much statistical difference in the HCV distribution with respect to Educational background and Occupation of subjects screened. However, the positivity rate among these two parameters has some important implications which warrant screening diabetic patients for HCV as this will reduce the increased risk of HCV infection in patients with T2DM and its transmission rate in our institutions of learning and possible work places, The observations recorded on Educational background and occupation of subjects screened are in agreement with the study of Ndako et al., [20] conducted earlier.

**Fig. 6:**
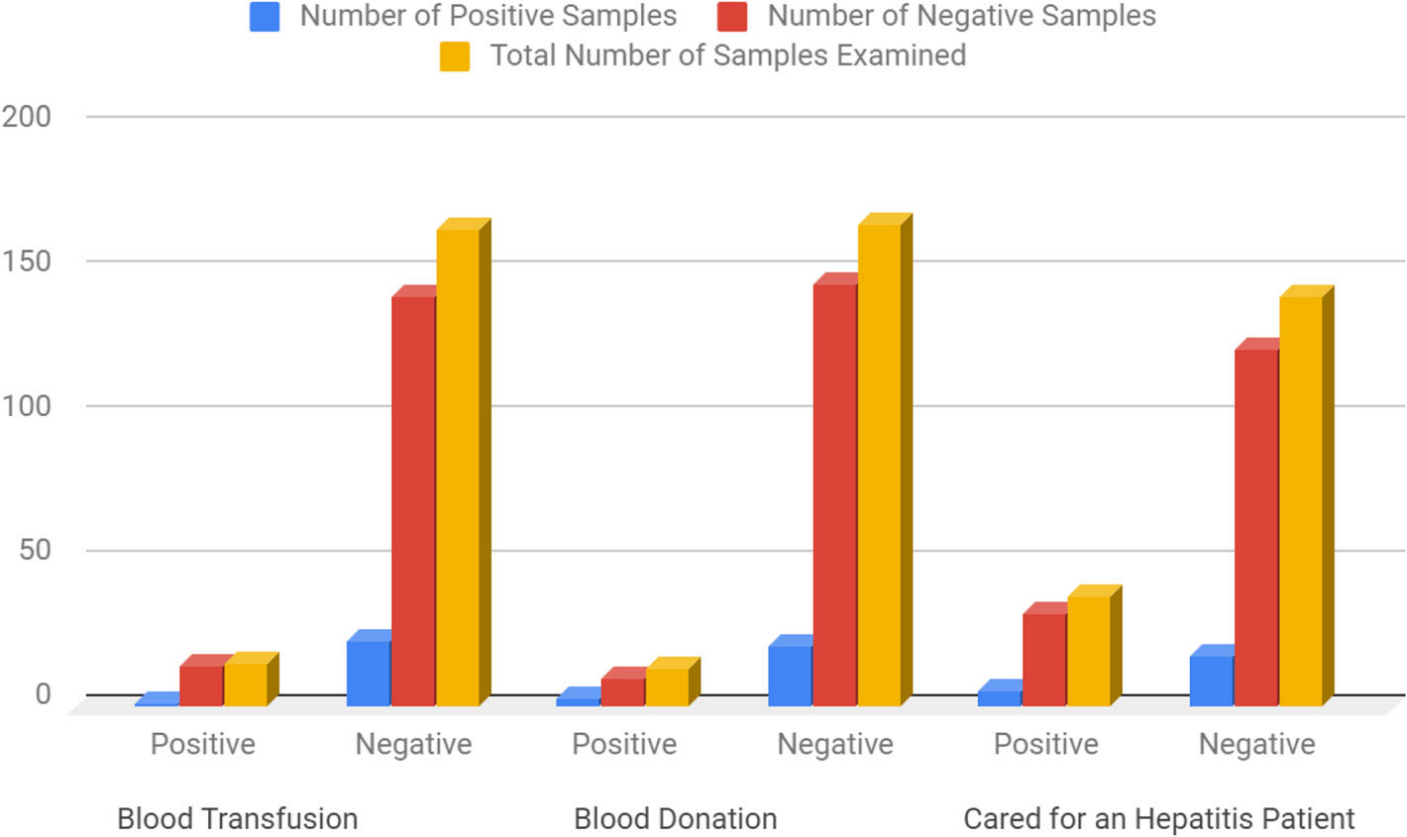
Distribution of Hepatitis C Virus based on Clinical Risk Factors

Considering clinical risk factors,(Table 8; Fig. 6) it was observed that regardless of the fact that HCV is a blood-borne virus, the sero-prevalence among individuals that had undergone blood transfusion or donation is of no statistical significant difference and this agrees with the work of Simo et al.*,* [28].Among subjects that had history of blood donation in this study, 3 (1.7%) were found to be positive to HCV. However, it was observed that blood and blood products are potential sources of transmission for HCV infection, Ndako et al., [19]. In the present study, sharing personal items such as sharp objects that may be contaminated with infected blood and tattoo practices were defined as HCV predictors. The risk of HCV continues to be a great occupational threat. Consequent upon which, blood transfusion was also identified as a predictor of HCV Infection [29].

High rate of positivity was equally observed in subjects screened for alcohol consumption, sharing of unsterilized objects also among subjects that had history of tribal marks or tattoo. Positivity rates ranged from 3.9, 2.2 and 2.8% respectively with no statistical significant difference.(Table 9). However, participants with family history of diabetes recorded a sero-prevalence of 6.7% to HCV infection, this report is similar to the work of Muller et al., [30] where the increased incidence of HCV was closely related with family history of diabetes mellitus. A significant difference was observed among participants with family history of diabetes mellitus compared to those without, [30, 31];(Table 10), this might be attributed to any of multiple sexual partners, transfusion of unscreened blood in hospitals, family history of related infections, risky behaviours such as alcohol intake and other potential unidentified routes of transmission which can only be discovered through advanced studies [32].

This study found that elevated liver enzymes; especially ALT has a direct relationship with seropositivity to HCV in the diabetic population studied, (Table 11), showing the relevance of checking for levels of liver transaminases as a screening test in diabetics. In a study by Mason, [33] more than 20% of diabetes patients with consistently elevated serum aminotransferases had evidence of HCV infection. It was also discovered that most of the anti-HCV positive diabetic patients presented with an abnormal liver function tests, a combination of hepatocellular and cholestasis pattern being the predominant biochemical alteration, Osi and Sanna., [34]. Elevation of ALT in hepatitis C positive diabetes patients in this study is unusually mild, with most having ALT level between one to two times upper limit of normal.

**Table 11:** Determination of Serum Alanine Aminotransferase on HCV patients

Age	No. Seropositive For HCV (%)	Normal ALT range (%)	Abnormal ALT range (%)
0–20	0(0%)	Not applicable	Not applicable
21–30	2(1.1%)	2(1.1%)	0(0%)
31–40	6(3.3%)	3(1.7%)	3(1.7%)
41–50	9(5.0%)	6(3.3%)	3(1.7%)
51–60	3(1.7%)	1(0.6%)	2(1.1%)
61–100	4 (2.2%)	2(1.1%)	2(1.1%)
Total	24(13.3%)	14(7.8%)	10(5.5%)

## Conclusion

The outcome of this study showed an increased risk of HCV infection in patients with T2DM, which warrants routine screening of diabetic subjects for HCV. Similarly, the study adds to the limited data on the subject available in this region and will help at increasing awareness regarding association of HCV and diabetes, which will further help to reduce morbidity and possible mortality associated with this comorbidity conditions in the long run. The need for prompt enlightenment of the general public on the threats of the co-infectious nature of HCV and diabetes is strongly advocated. In addition, it is important that health care workers, approach the settings of early diagnosis and management of this condition among infected persons to avert further complications among confirmed type 2 diabetics in the general population.

## Supplementary information

**Additional file 1.** (Questionnaire Sample).

## Data Availability

The datasets used and/or analyzed during the current study are available from the corresponding author on reasonable request.

## Abbreviations

HCV: Hepatitis C Virus
T2DM: Type 2 Diabetes Mellitus
ALT: Alanine Transaminases
ELISA: Enzyme-Linked Immunosorbent Assay
