# Supplementary material for: Studies on the prevalence of Hepatitis C virus infection in diabetic patients attending a tertiary health-care facility South-west Nigeria
Source: BMC Infect Dis. 2020 Sep 9;20:664. doi: 10.1186/s12879-020-05388-7 (PMC7488326; doi:10.1186/s12879-020-05388-7)
Supplement: Supplementary file 1 — Additional file 1. (Questionnaire Sample). [file 12879_2020_5388_MOESM1_ESM.docx]

**SAMPLE QUESTIONNAIRE**

**SCREENING FOR HCV ANTIBODIES AMONG DIABETIC PATIENTS ATTENDING FEDERAL TEACHING HOSPITAL IDO-EKITI**

**Please tick or circle the appropriate answer for each question as applicable.**

1. Sex: Male Female
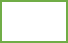

2. Age……………….
3. Marital status: Single
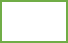
 Married
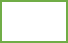
 Divorced
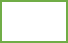

4. Marital relationship if married: Monogamy
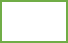
 Polygamy
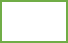
 Others
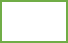

5. Educational Qualification: Primary
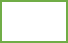
 Secondary
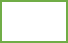
 Tertiary
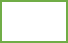
 No Education
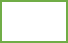

6. Occupation: Trading
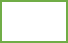
 Civil Servant
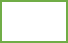
 Industry
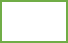
 Student
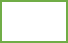

7. Social Status:………………………
8. Have you at any time in the past received blood transfusion Yes ( ) No ( )
9. If yes, how many times? a. (1) b. (2) c. (3) d.(4)
10. How many Pint(s)? a. (1) b. (2) c. (3) d. (4)
11. Has your spouse been transfused in the past? Yes ( ) No ( )
12. If yes, how many times? a. (1) b. (2) c. (3) d. (4)
13. How many Pint(s)? a. (1) b. (2) c. (3) d. (4)
14. Have you at any time donated blood? Yes ( ) No ( )
15. If yes, how many times? a. (1) b. (2) c. (3) d. (4)
16. How long have you been suffering from diabetes?..........................................................
17. What drug do you use in the mangement of this condition?..................................................
18. Has any of your family member suffered from:
19. Diabetes? Yes ( ) No ( )
20. Hepatitis? Yes ( ) No ( )
21. Do you take alcohol? Yes ( ) No ( )
22. If yes, how long have you been involve in alcohol taking? .........................................
23. Are you on any long term medication? Yes ( ) No ( )
24. If yes who prescribed the drugs and where? Self
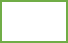
 Hospital
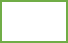

25. Do you have tribal marks/tattoos? Yes ( ) No ( )
26. How many sexual partner(s) have you had in the last 3-5 years……………………..
27. Have you had non-spousal sexual intercourse in the last 3-5 years? Yes ( ) No ( )
28. If yes, number of partner(s)……………………………………………………….
29. Do you share razor blade/Scissors/Nail cutters with your friends? Yes ( ) No ( )
30. Do you have a prior knowledge of Hepatitis Virus before? Yes ( ) No ( )
31. Have you seen/care for someone that has been infected with Hepatitis B or C virus before? Yes ( ) No ( )
32. Have you suffered from any of the Hepatitis virus before? Yes ( ) No ( )
33. Do you engage in risky behaviors such as: use of unsterilized sharp objects? Yes ( ) No ( )
